# Single-subunit RNA polymerases, KpnP, Ro45Iw, and CD23823, with precise terminal synthesis

**DOI:** 10.1016/j.jbc.2025.110359

**Published:** 2025-06-23

**Authors:** Haruka Takatsuki, Ryota Miyachi, Kaito Seo, Katsumi Hagino, Norikazu Ichihashi

**Affiliations:** 1 College of Arts and Science, The University of Tokyo, Meguro, Tokyo, Japan; 2 Department of Life Science, Graduate School of Arts and Science, The University of Tokyo, Meguro, Tokyo, Japan; 3 Earth-Life Science Institute, Institute of Science Tokyo, Tokyo, Japan; 4 Department of Bacteriology, University of Wisconsin-Madison, Madison, Wisconsin, USA; 5 Komaba Institute for Sciences, The University of Tokyo, Meguro, Tokyo, Japan; 6 Universal Biology Institute, The University of Tokyo, Meguro, Tokyo, Japan

**Keywords:** RNA polymerase, bacteriophage, promoter, RNA synthesis, viral polymerase, *in vitro* transcription, T7 RNA polymerase

## Abstract

T7 RNA polymerase (RNAP) is a highly active single-peptide polymerase that is widely used for *in vitro* RNA synthesis. T7 RNAP synthesizes RNA with a heterogeneous 3′-end, which sometimes causes problems. To date, some RNAPs (*e.g.*, KP34 and Syn5) that synthesize RNA with more precise 3′-ends have been reported. However, they have their own characteristics and thus do not fully substitute T7 RNAP. To increase the number of usable RNAP repertoires, we searched for other RNAPs and their promoters in the phage RNAP database. From the first screening of nine RNAPs, we selected three RNAPs, named KpnP, Ro45Iw, and CD23823, together with their promoter sequences. Characterization of recombinant RNAPs revealed that compared to T7, these three polymerases exhibited similar RNA synthesis activities but preferred a slightly lower temperature. Additionally, CD23823 exhibited a slightly higher salt tolerance. Deep sequencing of the 5′- and 3′-termini of the synthesized RNA revealed that the three RNAPs, particularly CD23823, produced more homogeneous termini than T7 RNAP. These results indicate that these previously uncharacterized RNAPs, especially CD23823, are useful for RNA synthesis that requires precise 5′- and 3′-end sequences.

T7 RNA polymerase (RNAP) and related bacteriophage RNAPs are single-subunit DNA-dependent RNAPs that are widely used in biotechnology for *in vitro* and *in vivo* RNA synthesis (1, 2, 3, 4, 5, 6, 7) owing to their high activity and promoter specificity (1, 8, 9). However, the T7 RNAP has some shortcomings. T7 RNAP is known to reduce its activity at high salt concentrations (10), which is disadvantageous for *in vitro* gene expression (11). T7 RNAP is also known to produce nonhomogeneous 3′-termini of RNA through the addition of nontemplated nucleotides after run-off transcription (12), self-templating reactions (13), and/or DNA terminus-initiated transcription (14). The latter two processes produce immunostimulatory double-stranded RNA (15), which causes serious problems when used as RNA medicine. Modification of the 3′-end of the DNA template reduces the 3′-end heterogeneity of RNA products (16). Several methods have been developed to address these problems. A mutant T7 RNAP produces fewer immunostimulatory products (17, 18). The addition of competing oligo DNA reduces the self-templating reaction (19).

Another possible solution to the problems of T7 RNAP is to find alternative RNAPs. In addition to T7 RNAP, a number of RNAPs of bacteriophages are stored in databases, whereas only a small number of polymerases have been characterized. KP34 RNAP from *Klebsiella* phage KP34 exhibit undetectable self-templated RNA terminus extension (20). Syn5 RNAP from marine cyanophages has been well-characterized. It has higher processivity, less frequent terminal nucleotide addition (20, 21), lower temperature preference (optimum at 24 °C), higher salt concentration preference (10), and higher modified nucleotide incorporation (22). VSW-3 RNAP from psychrophilic phages produces less dsRNA (23). EM1 RNAP found in metagenomic data is not prone to self-templated RNA synthesis (24). Generally, each polymerase has its own optimum conditions, such as temperature and salt conditions; therefore, searching for a greater variety of RNAPs is required for wider applications.

In this study, we searched for other RNAPs that have similar activity to T7 RNAP at 30 to 37 ˚C and produce a more homogeneous 3′-terminus of the RNA product after run-off transcription. As the first screening step, we tested nine polymerases unintentionally selected from a database and each predicted promoter in a cell-free transcription/translation system. We purified three recombinant polymerases from *Escherichia coli* and characterized their RNA synthesis activity at various temperatures and salt concentrations. We also analyzed the 5′- and 3′-terminal sequences of the RNA products using deep sequencing. From these analyses, we found three previously uncharacterized RNAPs (named KpnP, Ro45Iw, and CD23823) that had comparable activity at 30 °C to T7 RNAP but a more homogeneous terminus of the RNA product than T7 RNAP.

## Results

### Screening of phage RNAP

**Figure 1:**
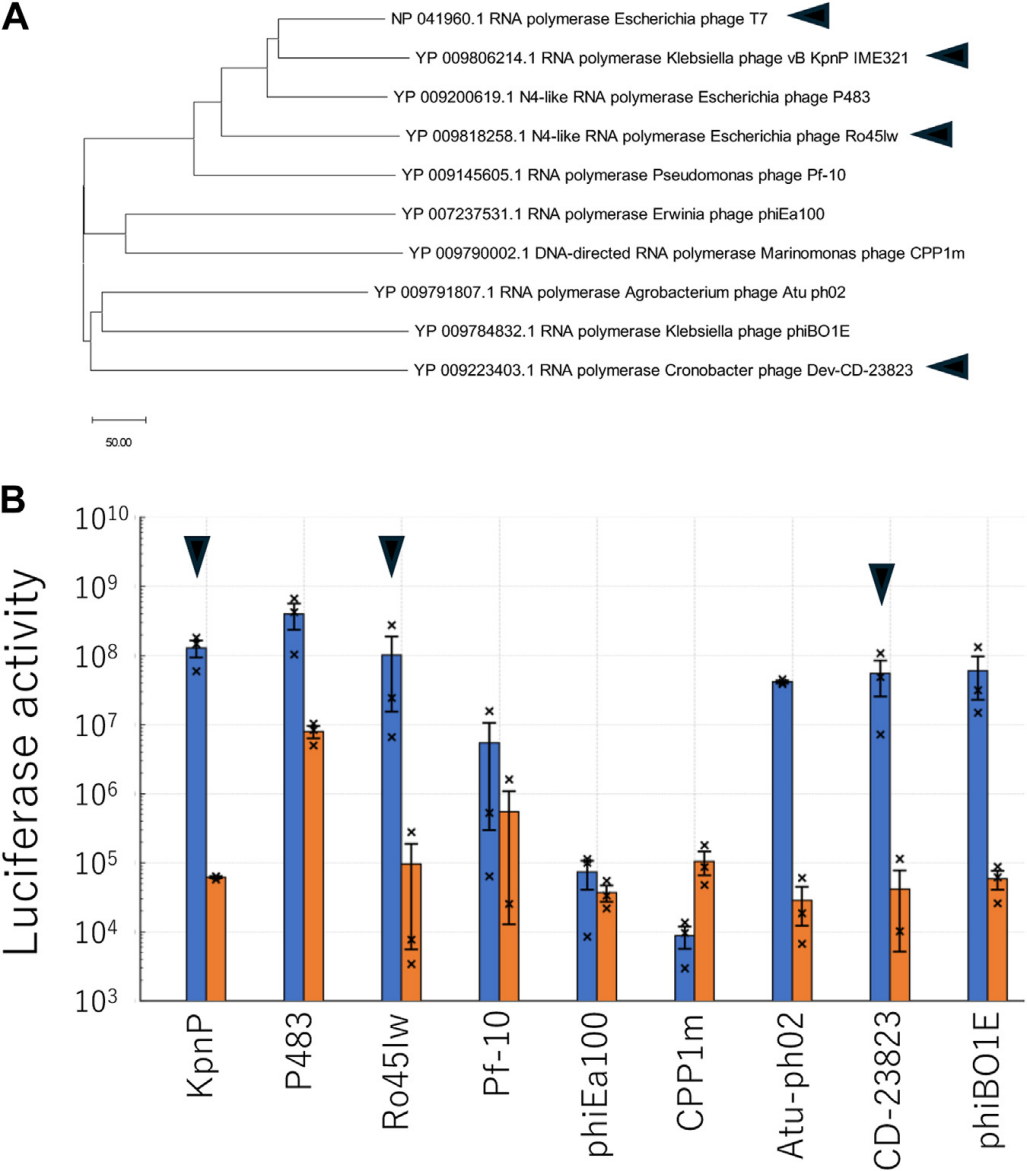
**First screening of phage RNAPs.***A*, phylogenetic analysis of the nine RNAPs selected from the NCBI Protein Database. The scale bar represents evolutionary distances computed based on the number of amino acid differences per sequence. *B*, evaluation of polymerase activity using co-expression experiments with a reporter gene. The reaction mixture contained luciferase under each promoter and a reconstituted transcription/translation system of *E. coli* with or without DNA fragments encoding each RNAP (RNAP DNA + or RNAP DNA-). After incubation at 37 °C for 2 h, luciferase activity was measured. The RNAP used in later experiments are indicated with *arrowheads*. Error bars represent standard errors (N = 3). Individual data points are technical replicates. RNAP, RNA polymerase.

To identify useful RNAPs with properties different from those of the widely used T7 RNAP, we obtained 38 phage RNAP sequences from the NCBI database and constructed a phylogenetic tree (Fig. S1). We then unintentionally selected nine RNAPs from different branches in the tree. A phylogenetic tree of the nine RNAPs is shown in Fig. 1*A*. We also estimated the promoter sequence for each polymerase, as described in the Experimental Procedures. For the first screening, we expressed each of the nine RNAPs in a reconstituted gene expression system of *E. coli* (PUREfrex 2.0) containing a firefly luciferase reporter gene under each estimated promoter. If the polymerases are expressed in their active form and the estimated promoter sequence is correct, luciferase activity should be detected depending on their expression. We detected luciferase activities for eight polymerases (KpnP, P483, Ro45Iw, Pf-10, phiEa100, Atu-ph02, CD23823, and phiBO1E) in the presence of the DNA fragment encoding each RNAP (RNAP DNA+) than the absence of it (RNAP DNA-) (Fig. 1*B*). Based on these results, we selected three polymerases (KpnP, Ro45Iw, and CD23823) for further analysis (black arrowheads). Additionally, we initially chose P483 for further analysis but failed the subsequent purification process; it was not expressed in its soluble form.

The luciferase activities significantly varied in the absence of RNAP DNA, depending on RNAPs. This variability is likely caused by different cross-reactivity of T7 RNA polymerase, which is included in the reconstituted gene expression system used in this experiment, with each promoter.

### Characterization of RNAPs

The three RNAP investigated in this study (KpnP, Ro45Iw, and CD23823) were obtained from bacteriophages Klebsiella phage vB KpnP IME321, *Escherichia* phage Ro45lw, and Cronobacter phage Dev-CD-23823, respectively. Sequence identity among these enzymes ranged from 23.7% to 73.4% (Fig. S2*A*), with a particularly low identity between CD23823 and the other RNAPs (23.7–25.4%). The amino acids in the catalytic center of T7 RNAP (Asp537, Lys631, and Asp812) (25) are conserved in all RNAPs examined (indicated with black arrowheads in Fig. S2*B*).

To characterize the selected RNAPs, we first purified each recombinant polymerase, together with T7, with a histidine tag from *E. coli* (Fig. S3). CD23823 RNAP was smaller than the others (T7, KpnP, and Ro45Iw), consistent with the gene size (T7:884 aa, KpnP: 907 aa, Ro45Iw: 894 aa, and CD23823:818 aa, without histidine tag).

**Figure 2:**
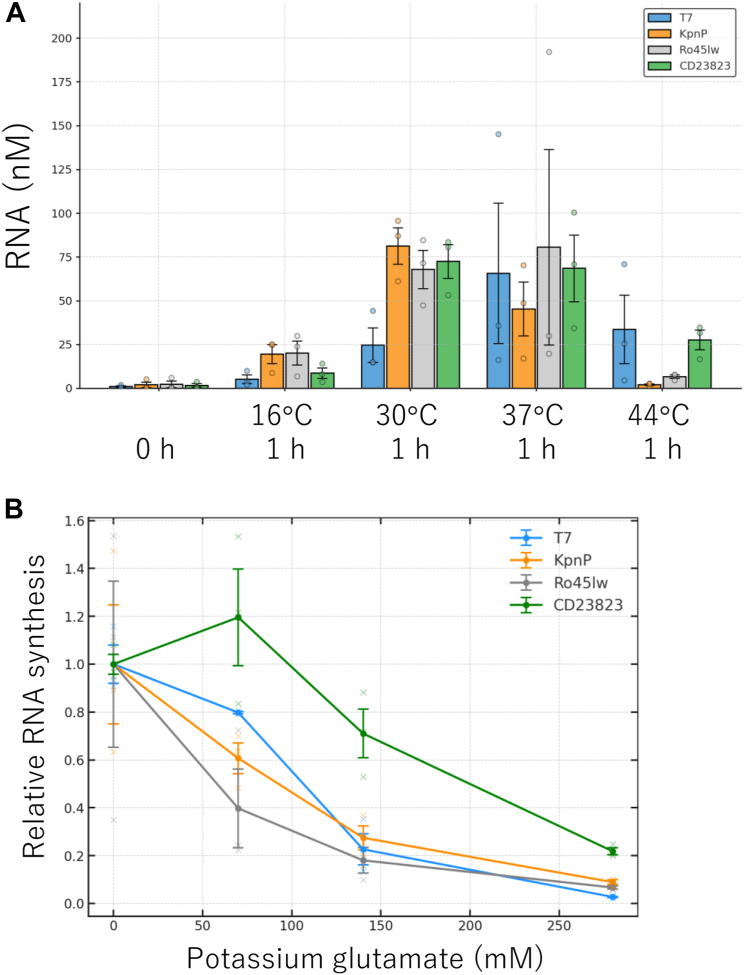
**Temperature and salt effects on RNA synthesis**. *A*, temperature effect. RNA synthesis reactions were conducted with 0.5 nM template DNA (encoding firefly luciferase gene) and 0.5 μM each RNAP at the indicated temperature for 1 h, and synthesized RNA was measured by quantitative PCR after reverse transcription. *B*, salt effect. RNA synthesis reactions were conducted with 0.5 nM template DNA and 0.5 μM each RNAP at 30 °C for 1 h at the indicated concentration of glutamate potassium. The synthesized RNA was quantified by quantitative PCR after reverse transcription. Error bars represent standard errors (N = 3). Individual data points are technical replicates. RNAP, RNA polymerase.

Using these recombinant polymerases, we compared their RNA synthesis activity at different temperatures. We incubated each polymerase and a DNA template containing each promoter at 16, 30, 37, and 44 °C for 1 h, and the RNA concentration was measured by quantitative PCR after reverse transcription (Fig. 2*A*). We found that RNA synthesis varied depending on the temperature. RNA syntheses were similar for all polymerases at 37 °C, but the RNA synthesis by the three new polymerases (KpnP, Ro45Iw, and CD23823) was higher than that by T7 at 30 °C, indicating that the optimum temperatures of these new RNAPs are slightly lower than those of T7 RNAPs. Additionally, we checked the size of the RNA synthesized at 37 °C by agarose gel electrophoresis and found that the sizes were the same as expected (Fig. S4).

We next examined the salt tolerance of RNA synthesis because T7 RNAP activity decreases at higher salt concentrations (10), which causes problems when combining *in vitro* transcription with translation (11). When salt (potassium glutamate) was added to the reaction mixture, RNA synthesis decreased for T7, KpnP, and Ro45Iw, while it increased at 70 mM potassium glutamate for CD23823 (Fig. 2*B*), indicating that CD23823 is slightly more salt-tolerant than the other polymerases.

### Characterization of 5′-terminal sequence of the synthesized RNA

**Figure 3:**
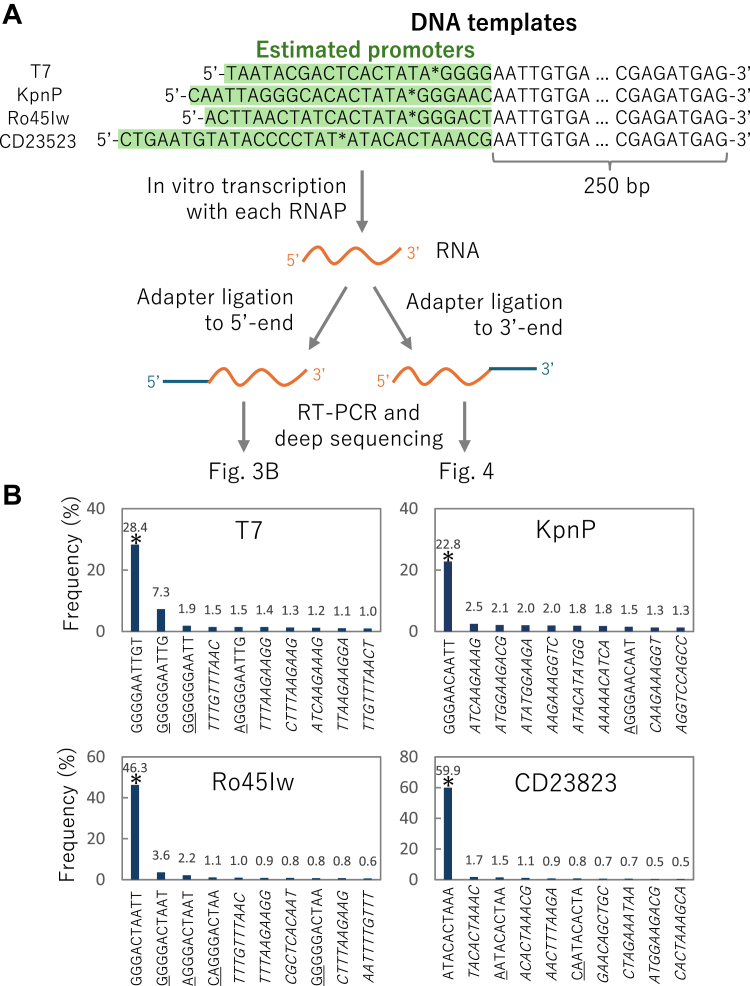
**Analysis of 5′-termini of the synthesized RNA**. *A*, scheme of analysis. Each DNA template was transcribed by each RNAP. Estimated promoter sequences are highlighted in *green*. The most frequent +1 sites found in B are indicated by *asterisks*. After ligating an adaptor to the 5′- or 3′-end of the RNA, the RNA was reverse-transcribed and subjected to deep sequencing. *B*, frequency of the top 10 sequences of the 5′-end of the transcript. The most frequent sequences are indicated with *asterisks*. The additional nucleotides that do not exist in the template are underlined. Sequences that were not found around the promoter are shown in *italics*. The read numbers were 715,461, 528,652, 669,119, and 716,850 for T7, KpnP, Ro45Iw, and CD23823, respectively. RNAP, RNA polymerase.

**Figure 4:**
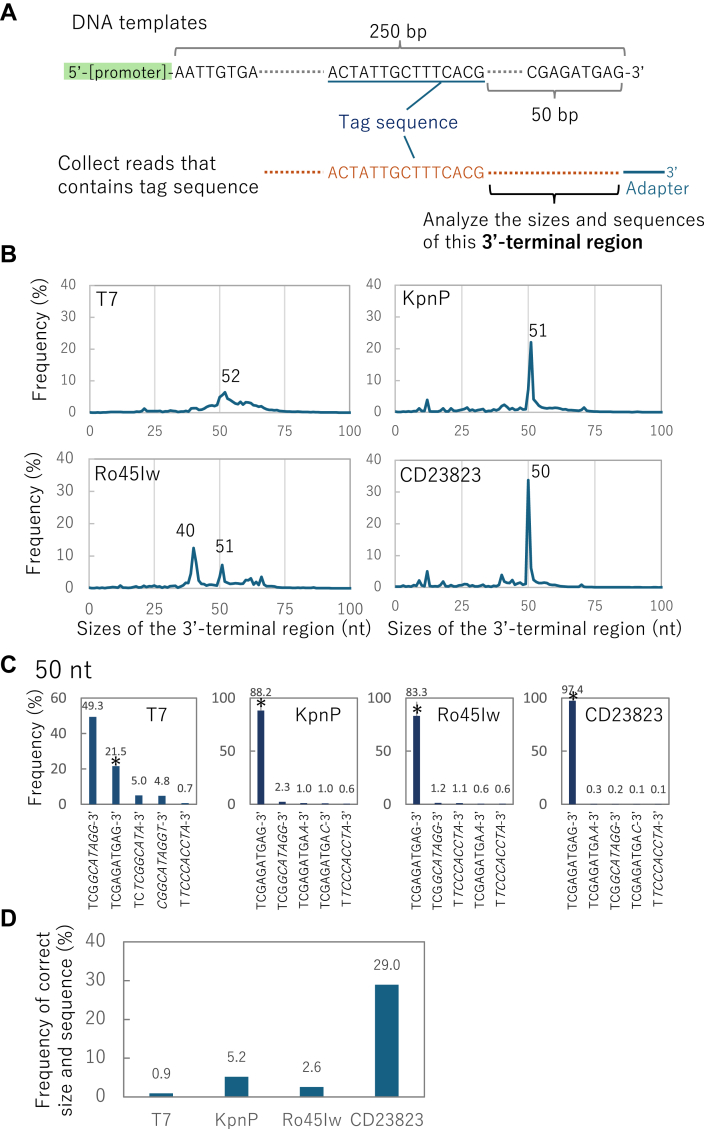
**Analysis of 3′-termini of the synthesized RNA.***A*, analysis scheme. Each synthesized RNA was attached to an adaptor at the 3′-end (Fig. 3*A*) and analyzed. From the sequence data, reads containing a tag sequence (5′-ACTATTGCTTTCACG), which was located at the-50 bp position from the 3′-end of the template DNA, were collected. The downstream sequence of the tag sequence was then analyzed. The read numbers analyzed were 236,579, 155,240, 348,559, and 111,761 for T7, KpnP, Ro45Iw, and CD23823, respectively. *B*, size distribution of the 3′-terminal region of each synthesized RNA. If transcription stops precisely at the terminus of the template, the size should be 50 nt. Smaller or longer sizes indicate an interruption or unexpected elongation of transcription, respectively. The sizes of the major peaks are also shown. *C*, *top*-5 sequences of the 3′-terminal sequences at 50 nt. Sequences that exactly match the 3′-end of the DNA template sequence are marked with *asterisks*. The additional nucleotides that do not exist in the template are *underlined*. Sequences that were not found around the terminus of the DNA template are shown in *italics*. *D*, frequencies of RNA with the same size and sequence as the 3′-terminus of the DNA template. To calculate the frequencies, the frequency of the correct size (*i.e.*, 50 nt) was multiplied by the ratio of correct sequences shown in *C* (*asterisks*).

To determine the +1 initiation site for each RNAP, we analyzed the 5′-terminal sequence of the synthesized RNA. We conducted run-off transcription of short RNA (expectedly 250 nt) using an RNAP and a DNA template at 37 °C for 4 h (Fig. 3*A*). Then, an adaptor oligo RNA was ligated to the 5′-end of the transcript, reverse transcribed, PCR-amplified, and subjected to deep sequencing. In addition, we ligated another adaptor oligo RNA to the 3′-end of the transcript for 3′-terminal sequence analysis, which is shown in Fig. 4.

Fig. 3*B* shows the top-10 most frequent 5′-terminal sequences. The most frequent sequences for each RNAP are indicated by asterisks. Based on this result, the +1 site was estimated and is indicated by asterisks in Fig. 3*A*. For T7 RNAP, the +1 site was G, which is consistent with the known initiation site (26). The +1 site of KpnP and Ro45Iw RNAP were also G, whereas that of CD23823 was A.

The frequency of transcripts that started from each +1 site varied depending on the RNAPs. The frequencies for T7 and KpnP polymerases were approximately 28% and 22%, respectively, whereas they were higher for Ro45Iw (46%) and CD23823 (60%). A substantial fraction (∼9%) of RNA synthesized by T7 RNAP contained additional Gs at the 5′-terminus. These data indicated that the transcripts produced by Ro45Iw and CD23823 had a more homogeneous 5′-end sequence than those produced by T7 RNAP. It should be noted that many minor 5′-end sequences unrelated to the promoter sequence were detected in this assay (shown in italics in Fig. 3*B*). These 5′-end sequences could be produced by nonstandard transcriptional initiation and RNA degradation during *in vitro* transcription and subsequent procedures.

### Characterization of 3′-terminal sequences of synthesized RNA

We investigated the 3′-end sequences of the RNA synthesized by RNAPs. As shown in Fig. 3*A*, after ligating the adaptor oligonucleotide at the 3′-end of short RNA transcripts, the 3′-terminal sequences were analyzed. To investigate the variety of 3′-terminal sequences, we first compared the size of the 3′-terminal sequence to that expected from run-off transcription of the template DNA. The analysis scheme is shown in Fig. 4*A*. We first collected reads that contained a tag sequence (ACTATTGCTTTCACG) located 50 nt from the 3′-end of the DNA template. We then analyzed the size of the downstream sequence (3′-end sequence) between the tag and the adaptor. If the run-off transcription occurs correctly, the size should be 50 nt.

The size distributions of the 3′-end sequences differed among the RNAPs. T7 RNAP exhibited a relatively broad peak, consistent with the results of a previous study (13). The major peak was at 52 nt, which was 2 nt longer than the template size. Ro45Iw exhibited multiple sharp peaks, one of which was located at 51 nt. KpnP and CD23823 exhibited major sharp peaks at 51 nt and 50 nt, respectively. Next, we compared the frequencies of synthesized RNA with the same 3′-end sequences as the DNA template (*i.e.*, run-off transcription occurred correctly). To calculate this value, we should multiply the frequencies of the correct 5′-end sequence size (*i.e.*, 50 nt) by the ratio of the correct sequence at 50 nt. Figure 4*C* represents top-5 most frequent 3′-sequences at 50 nt for each RNAP. The frequencies of the correct sequences that matched the 3′-end of the DNA template (indicated with asterisks) were 22.5%, 88.2%, 83.3%, and 97.4% for T7, KpnP, Ro45Iw, and CD23823, respectively. By multiplying these values by the frequencies of 50 nt sequencies (shown in Fig. 4*B*), we calculated the frequencies of the correct run-off transcription product for each RNAP (Fig. 4*D*). The value was as low as 0.93% for T7 RNAP and higher for the other polymerases (5.2%, 2.6%, and 29% for KpnP, Ro45Iw, and CD23823, respectively), indicating that RNA with relatively homogenous 3′-ends can be obtained using these RNAPs.

We also investigated the top-5 most frequent 3′-end sequences at 51 and 52 nt of the 3′-end sequences (Fig. S5). We found that most of the sequence at the peak of T7 (52 nt) included sequences not found in the DNA template, such as TCTCGGCATAGG (indicated with a black arrowhead), which can be produced by a self-templating reaction after transcription interruption, as described in the Discussion section. The majority of the 3′-end sequences of transcripts produced by KpnP and Ro45Iw at their peaks (51 nt) had one additional nontemplated nucleotide at the 3′-end (indicated with white arrowheads in Fig. S5).

### Essential region for each promoter sequence

**Figure 5:**
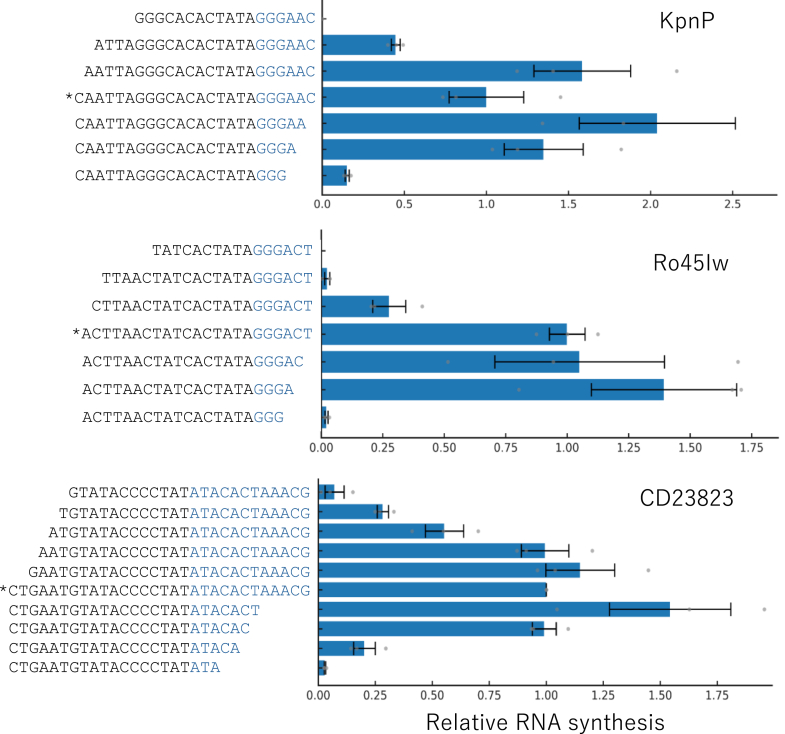
**Determination of the essential region of the promoter sequences**. RNA synthesis reactions were conducted with 0.5 nM template DNA with various promoter sequences and 0.5 μM each RNAP at 30 °C for 1 h, and synthesized RNA was measured by quantitative PCR after reverse transcription. The synthesized RNA concentrations are shown as relative values to the original estimated promoter, indicated with *asterisks*. Error bars represent standard errors (N = 3). Individual data points are technical replicates. Sequences after the +1 site are colored in *blue*.

To determine the essential region for each estimated promoter for the three new RNAPs, we constructed various promoters and compared their RNA synthesis efficiency (Fig. 5). The initially estimated promoter sequences are indicated with asterisks. For the KpnP promoter (CAATTAGGGCACACTATAGGGAAC), the 5′-terminal C and 3′-terminal AC were removed without decreasing RNA synthesis. For Ro45Iw (ACTTAACTATCACTATAGGGACT), the 3′-terminal CT was removed without decreasing RNA synthesis. For the CD23823 promoter (CTGAATGTATACCCCTATATACACTAAACG), the 5′-terminal CTG and 3′-terminal TAAACG were removed without decreasing RNA synthesis. Based on these results, the minimum promoter sequences that sustained a higher RNA synthesis ability were 5′-AATTAGGGCACACTATAGGGA for KpnP, 5′-ACTTAACTATCACTATAGGGA for Ro45Iw, and 5′-AATGTATACCCCTATATACAC for CD23823 (+1 sites are underlined).

Recently, consensus promoter sequences were investigated using a large number of phage genome sequences (27). Based on this report, the promoter sequences of KpnP (5′-AATTAGGGCACACTATAGGGA) and Ro45Iw (5′-ACTTAACTATCACTATAGGGA) were categorized as T7-like promoters, whereas the promoter for CD23823 (5′-AATGTATACCCCTATATACAC) was not similar to the reported promoter categories.

## Discussion

In this study, we searched for RNAPs that are useful for *in vitro* transcription from the phage RNAP database and identified three previously uncharacterized RNAPs (KpnP, Ro45Iw, and CD23823) and their promoters. The characterization of RNA synthesis ability showed that these polymerases exhibited similar RNA synthesis activities but worked more efficiently at lower temperatures (*e.g.*, at 30 °C) than T7 RNAPs (Fig. 2*A*). One of the polymerases (CD23823) exhibited slightly higher salt tolerance than T7 (Fig. 2*B*). The 5′-terminal sequence analysis of each transcript revealed that the RNA synthesized by Ro45Iw and CD23823 was more homogeneous than that synthesized by the other RNAPs (Fig. 3). The 3′-terminal sequence analysis of each transcript revealed that a higher frequency of CD23823 transcripts matched the 3′-end of the template DNA. These results indicate that more homogeneous RNA transcripts can be synthesized using CD23823 RNAP than using T7 RNAP. RNA synthesis with a precise terminus is important for *in vitro* synthesis of tRNA or rRNA. Previously, the termini of these RNA were prepared using self-cleaving ribozymes after *in vitro* transcription (28). The precise terminal synthesis ability of these RNAPs, together with previously reported RNAPs (*e.g.*, syn5, KP34, and VSW-3), provides useful tools for wider applications.

The 3′-terminal sequence of the RNA synthesized by T7 RNAP is highly heterogeneous. Only a small fraction (0.93%) of the sequences matched the termini of the DNA template (Fig. 4*D*). The major peak of the 3′-end sequence was 52 nt (Fig. 4*B*). The most frequent sequences of this size (Fig. S5, black arrowheads) were not found in the template strand but was found in the complementary sequence in the DNA template (Fig. S6). Such unusual RNA termini can be produced by a self-templating reaction that often occurs during transcription by T7 RNAP (13). Self-templating polymerization is the process by which a polymerase restarts transcription from the 3′-end of RNA, using RNA as a template. The unexpected 3′-terminal sequence by T7 RNAP can be explained if the transcript stops at the −10 position from the 3′-end of the template, where the hairpin structure is formed, as described in the next paragraph, and RNA synthesis restarts from the 3′-end of the transcript that forms a hairpin structure (see the image in Fig. S6 bottom). For KpnP, Ro45Iw, and CD23823 RNAPs, we did not observe self-templating products as major transcripts. The self-templating transcription abilities of these new polymerases were expectedly lower than that of T7 RNAP, which can explain the relatively sharp peaks in the size distribution of the 3′-terminal sequences for these new polymerases (Fig. 4*B*).

The 3′-terminal sequences of transcripts by Ro45Iw exhibited a major peak at 40 nt, which was different from other RNAPs (Fig. 4*B*). Most (90%) of the 40 nt sequences were TACCTATGCCGAGTAT. This sequence exists in the DNA template at −10 position from the terminus. Based on these results, we believe that the peak at 40 nt was caused by an unexpected termination at the −10 position from the 3′-terminus. The sequence of this position is shown in Fig. S7. RNA folding prediction (29) revealed that this region forms a hairpin structure. Ro45Iw polymerase might have weaker processivity than other polymerases and thus stop polymerization during this hairpin structure.

These novel RNAPs have potential for use in the production of mRNA medicine. However, critical aspects remain to be elucidated, such as their compatibility with standard capping strategies (cotranscriptional or post-transcriptional), efficiency in synthesizing poly A tails, and capacity to incorporate modified nucleotides. While these aspects are beyond the scope of the current study, investigating them and identifying more suitable enzyme mutants are crucial future research directions.

## Experimental procedures

### Sequence analysis

Amino acid sequences of the bacteriophage RNAPs (38 sequences in total) were obtained from the NCBI Protein database. After alignment using MUSCLE (30), a phylogenetic tree was constructed using the Neighbor-Joining method (31). To obtain the promoter sequence for each RNAP, we first collected genome sequences from the NCBI database. Second, candidates for each promoter sequence were obtained using software (PhagePromoter Ver 0.1.0) (32). Third, we chose one candidate that appeared multiple times in the genome and was located upstream of the open reading frames. The final promoter candidates are shown in the Supplementary Data.

### Plasmid preparation

Expression plasmids encoding the nine bacteriophage RNAPs were synthesized using an artificial gene synthesis service (Twist Bioscience). The codon usage of the polymerase genes was optimized for *E. coli* using GENEius software from Eurofins, and the genes were then inserted into the pET21a vector with the T7 promoter. The plasmid sequences are shown in the Supporting Information. For T7 RNAP, we used pQE-T7RNAP reported previously (33). The plasmids used for recombinant polymerase purification were constructed by inserting a histidine tag at the N terminus using PCR. The resultant plasmid sequences are shown in the Supporting Information. A plasmid encoding firefly luciferase under the T7 promoter (pEX-Fluc) was previously constructed (34).

### First screening using PURE system

DNA fragments used for RNAP expression were prepared by PCR using primers 1 and 2, and each plasmid was used as a template. The DNA fragments used for T7 RNAP expression were prepared by PCR using primers 3 and 4 and each pQE-T7RNAP template. DNA fragments encoding firefly luciferase under each promoter candidate were prepared by PCR using primers 2 to 15 and pEX-Fluc as a template. Primer combinations and sequences are shown in the Supporting Information. The PCR products were purified using QIAquick DNA column (QIAGEN) before use.

The DNA fragment encoding luciferase under each promoter candidate (0.5 nM) and that encoding each RNAP (5 nM) were incubated in PUREfrex 2.0 (GeneFrontier), which include T7 RNAP, at 37 °C for 2 h. The luciferase activity of the mixtures was measured using Luciferase Assay Reagent (Promega) and a luminometer (GloMax, Promega).

### Purification of recombinant RNAPs

For purification of T7, KpnP, Ro45Iw, and CD23823 RNAPs, histidine-tag sequence (AGAGGATCGCATCACCATCACCATCACGGATCC) was inserted after the first methionine of the corresponding plasmids by PCR. The resultant plasmids and pQE-T7RNAP for T7 RNAP were introduced into an *E. coli* strain (Rosetta(DE3)pLysS). The strains were cultured at 37 °C until late log phage (*A*_600_ = 0.6–1). Next, 1 mM IPTG was added and incubated for 3 h. After harvesting, the cells were lysed by sonication, and the target protein was purified using a HisTrap column (Cytiva), as described previously (35). The purified proteins were stored at −80 °C in a buffer containing 20 mM Hepes-KOH (pH 7.6), 0.5 mM dithiothreitol, 0.05 mM EDTA, 100 mM NaCl, and 50% glycerol. Protein concentrations were determined based on A280.

### RNA synthesis assay of the recombinant RNAPs

The reaction mixture contained each recombinant RNAP (0.5 μM), the DNA fragment encoding luciferase downstream of each promoter (0.5 nM), Tris-HCl (40 mM, pH8.0), MgCl_2_ (8 mM), spermidine (2 mM), dithiothreitol (5 mM), NTP (2 mM each), and RNasin Plus Ribonuclease Inhibitor (0.4 unit, Promega). For dilution of RNAPs, a dilution buffer containing 20 mM Hepes-KOH (pH 7.6), 0.5 mM dithiothreitol, 0.05 mM EDTA, 100 mM NaCl, 40% glycerol, and 1 mg/ml BSA was used. The inclusion of BSA was important for maintaining RNAP activity. The mixtures were incubated at the indicated temperatures for the indicated time periods. RNA concentration was measured by quantitative PCR after reverse transcription using primers 16 and 17 and the One Step PrimeScript PLUS RT-PCR Kit (Takara). The absolute value of RNA concentration was estimated by drawing a standard curve using known concentrations of luciferase RNA prepared by *in vitro* transcription.

### Terminal sequencing of synthesized RNA

DNA fragments encoding a short RNA (250 nt, a former part of the luciferase template) under each promoter were prepared by PCR using the primers listed in the Supporting Information. The PCR products were purified using a QIAquick DNA column (QIAGEN). Using these short DNA fragments (2.5 nM) as templates, each RNA was synthesized using each purified RNAP (2.5 μM) at 37 °C for 4 h as described above. The synthesized RNA was purified using an RNeasy column (QIAGEN).

For 5′-end analysis, an aliquot of the purified RNA was treated with RppH (New England Biolabs) according to the manufacturer’s instructions to dephosphorylate the 5′-terminus and then ligated with an RNA oligo (5′-CCAGGCUUAUGGCAGUCACA, 1.44 mM) at the 5′-terminus using T4 RNA ligase (Takara) with 25% polyethylene glycol at 16 °C for 16 h according to the manufacturer’s instructions. After purification of the RNA using an RNeasy column (QIAGEN), the 5′-termini were reverse-transcribed using PrimeScript RTase (Takara) and primer 19 at 42 °C for 60 min, followed by 70 °C for 15 min, according to the manufacturer’s instructions. Then, the cDNA was PCR-amplified using primers 19 and 21 to 24 with the combinations shown in the Supporting Information. The DNA corresponding to the target size was purified by gel extraction, further PCR amplified using the same primers, purified again using a QIAquick column (QIAGEN), and subjected to DNB-SEQ analysis.

For 3′-end analysis, an aliquot of the purified RNA was ligated with an RNA oligo (5′-CACGAGCGUUUUCCCACCUA, 1.44 mM), which is phosphorylated at the 5′-end, at the 3′-terminus using T4 RNA ligase (Takara) with 25% polyethylene glycol at 16 °C for 16 h according to the manufacturer’s instructions. After purifying the RNA using RNeasy column (QIAGEN), 3′-temini was reverse transcribed using PrimeScript RTase (Takara) and primer 21 to 24 at 42 °C for 60 min, followed by 70 °C for 15 min, according to the manufacturer’s instructions. Then, the cDNA was PCR-amplified using primers 25 and 26 to 29 with the combinations shown in Supporting Information. The DNA corresponding to the target size was purified by gel extraction, further PCR-amplified using the same primers, purified using a QIAquick column (QIAGEN), and subjected to DNB-SEQ analysis.

### Analysis of the terminal sequence data

Sequence data were analyzed using custom Python scripts. For 5′-terminal analysis, we collected reads that contained the adaptor sequence (ATGGCAGTCACA) followed by any 20 nucleotides for each RNAP data. Next, we analyzed the 20-nucleotide variable region, which corresponds to the 5′-region of RNA. For the 3′-terminal analysis, we collected reads that contained the internal tag sequence (ACTATTGCTTTCACG), followed by an arbitrary sequence and the adaptor sequence (CACGAGCGTTTT) for each RNAP read. The internal arbitrary sequences, which should be 50 nt if the run-off transcription occurs correctly, were analyzed as the 3′-terminal region in Fig. 4. To determine the relative abundance of each 5′-terminal sequence, the number of reads for each sequence was normalized to the total number of analyzed reads.

## Data availability

All the data are contained in the manuscript and Supporting Information.

## Supporting information

This article contains supporting information.

## Conflict of interest

The authors declare that they have no conflicts of interest regarding the content of this article.
