# Supplementary material for: Single-subunit RNA polymerases, KpnP, Ro45Iw, and CD23823, with precise terminal synthesis
Source: J Biol Chem. 2025 Jun 23;301(7):110359. doi: 10.1016/j.jbc.2025.110359 (PMC12272871; doi:10.1016/j.jbc.2025.110359)
Supplement: Supporting_Information [file mmc1.pdf]

Supporting Information

Single-subunit RNA polymerases, KpnP, Ro45Iw, and CD23823, with precise terminal synthesis

Haruka Takatsuki, Ryota Miyachi, Kaito Seo, Katsumi Hagino, Norikazu Ichihashi

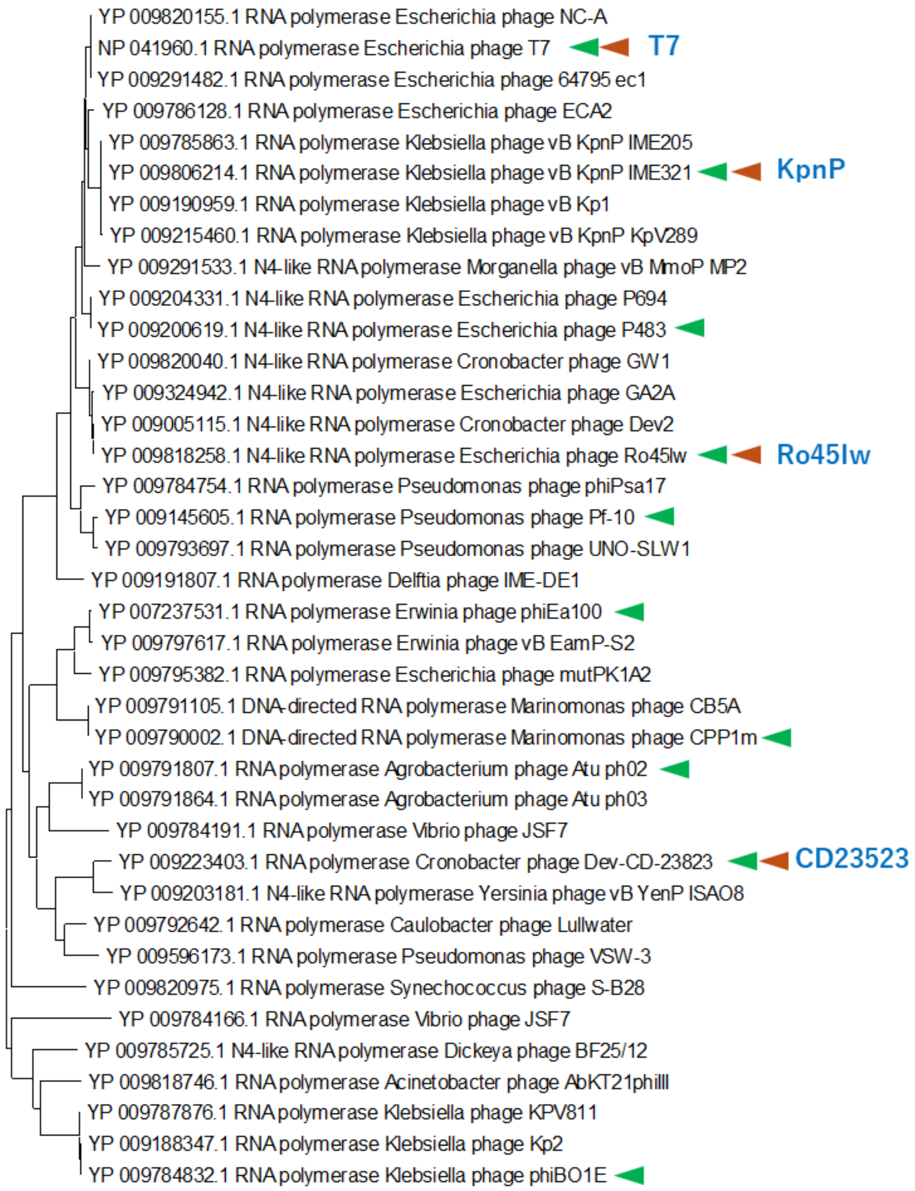

Figure S1. Phylogenetic tree of all analyzed phage RNAPs.

The RNAPs used for the first screening in Fig. 1 are indicated by green arrowheads. Purified RNAPs are indicated by arrowheads.

A

	KpnP	Ro45lw	CD-23823
T7	73.4	61.6	25.4
KpnP		59.0	24.4
Ro45lw			23.7

B

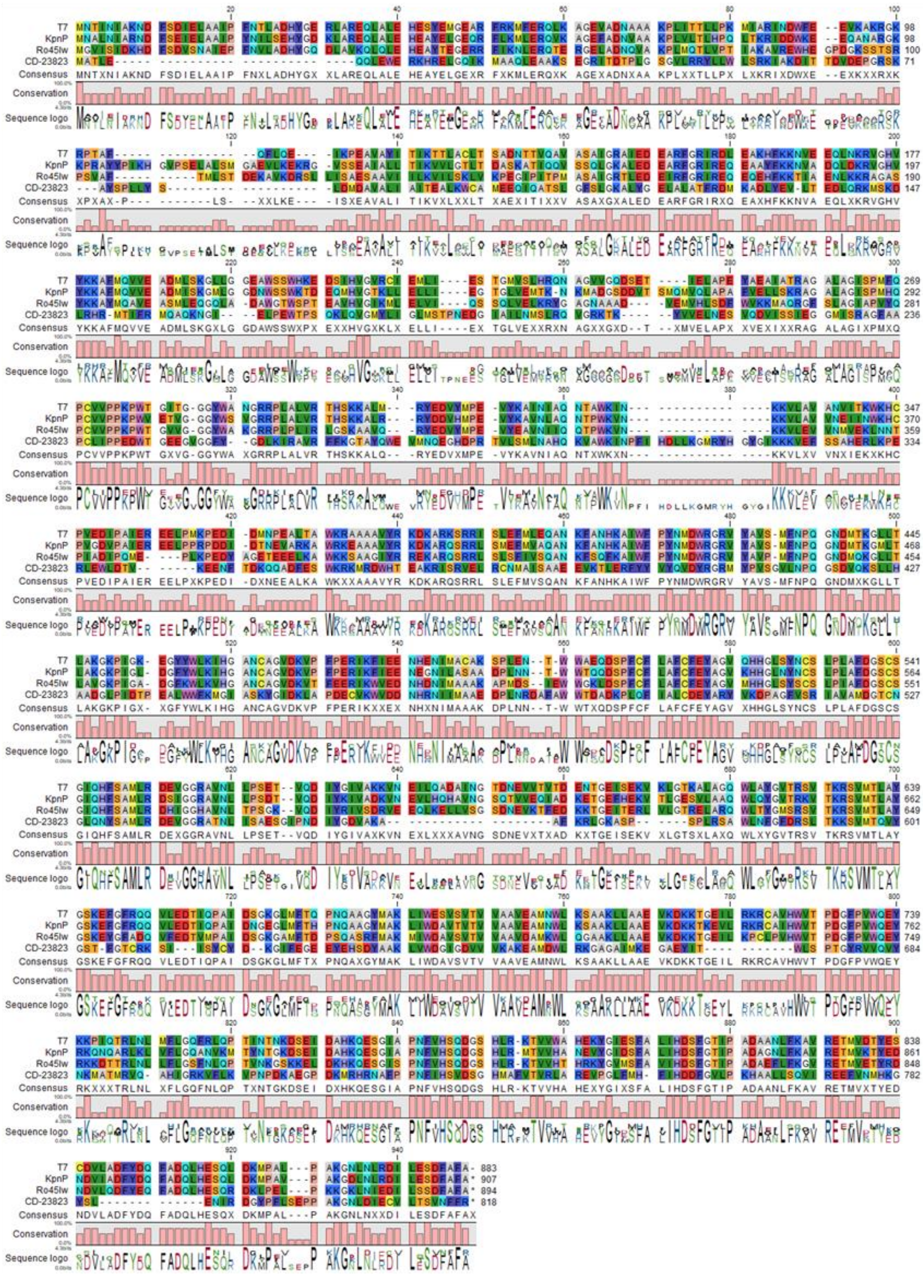

Figure S2. Amino acid sequence alignment of RNA polymerases.

(A) Percent identity of each pair of RNAPs. (B) Alignment of RNAPs constructed using CLC main workbench (QIAGEN).

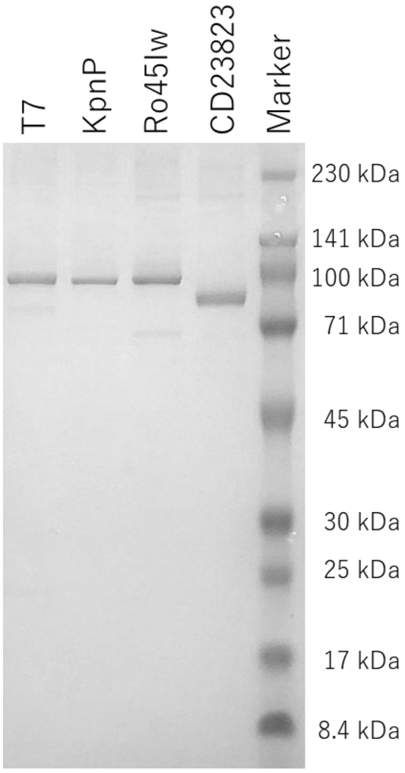

Figure S3. SDS-polyacrylamide-gel electrophoresis of the recombinant RNAPs

Proteins were separated on a 10-20% gradient gel and stained with Coomassie Brilliant Blue R-250.

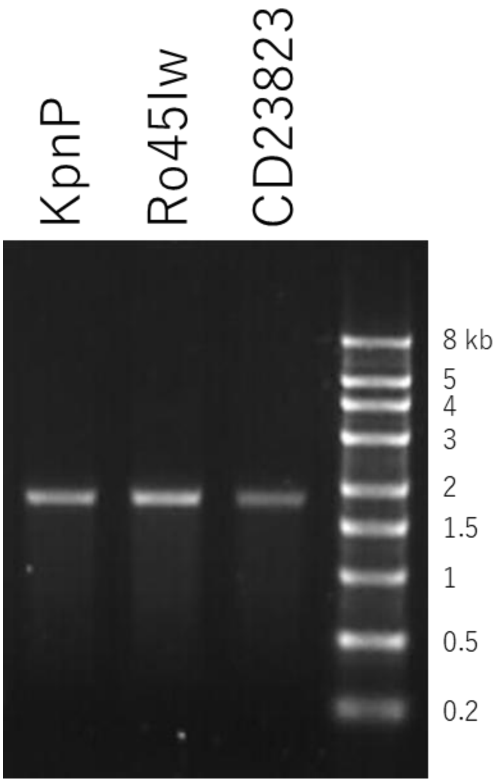

Figure S4. Agarose-gel analysis of RNA transcripts

RNA synthesis reactions were conducted with 0.5 nM template DNA (expected RNA size of 1.8 kb) and 0.5 μM each RNAP at 37°C for 1 h. The synthesized RNA was subjected to 1% denatured agarose gel electrophoresis using a size marker.

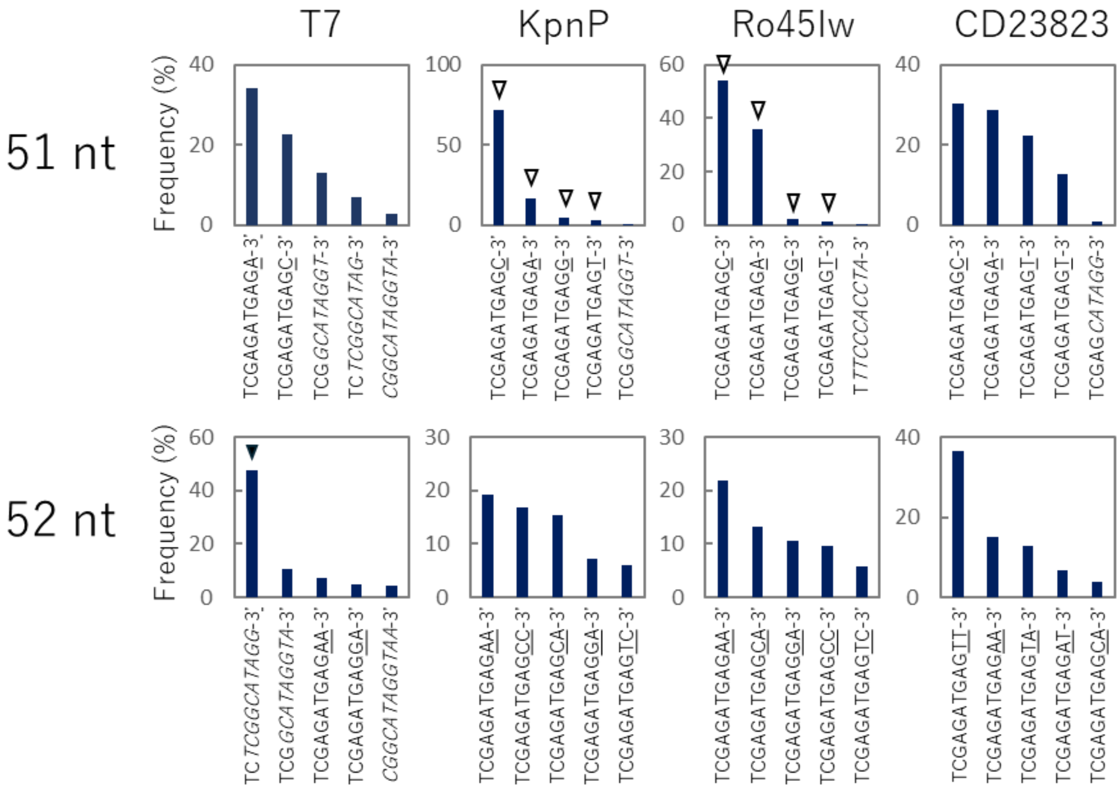

Figure S5. 3'-terminal sequences of each size of the synthesized RNA

Top-5 sequences of 3'-terminal sequences at 51 and 52 nt, shown in Fig. 4, were analyzed. Sequences that contain additional nucleotides at the 3'-end of the DNA template sequence are marked with white arrowheads only for KpnP and Ro45Iw at 51 nt. The most frequent sequence at the peak for T7 RNAP mentioned in the main text is indicated by the black arrowhead. The additional nucleotides that do not exist in the template are underlined. Sequences not found around the 3'-terminus of the DNA template are shown in italics.

3’-terminal sequence of DNA template

5'-[200 nt]-GATGCGCATATCGAGGTCAATATTACCTATGCCGAGTATTTCGAGATGAG-3'

Most frequent 3’-terminal sequence of transcript by T7 RNA polymerase.

5'-[200 nt]-GATGCGCATATCGAGGTCAATATTACCTATGCCGAGTATTTCTCGGCATAGG-3'

Self-templating reaction that produce the 3’-terminal sequence of the transcript (“T” were converted to “U”)

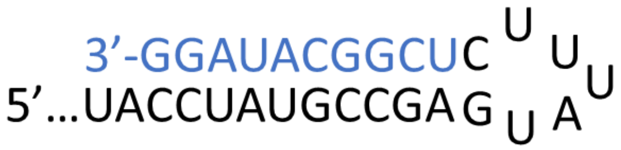

Figure S6. Possible mechanisms of unexpected 3’-terminal sequences in the synthesized RNA by T7 RNAP: self-templating polymerization

If the transcription stops at the-10 position from the 3’-end of the DNA template and is restarted using the product RNA as a template, the observed terminal sequence (TCGGCATAGG-3’ shown in blue) can be produced.

5'-[200 nt]-
GAUGCGCAUAUCGAGGUCAAUAUUACCUAUGCC
GAGUAUUUCGAGAUGAG-3'

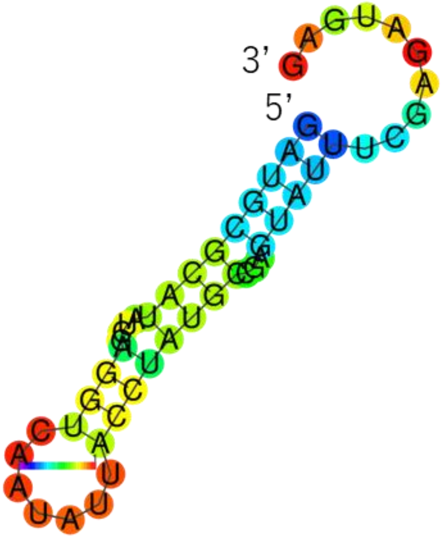

Figure S7. Sequence and possible RNA structure at the 3’-terminus of the synthesized RNA.

The expected 3’-terminal sequence of the synthesized RNA and its predicted structure are shown. The most frequent 3’-terminal sequence observed for Ro45Iw at 40 nt (Fig. 4B), is underlined. The RNA structure was estimated using the Vienna RNA fold, and the minimum free energy structure is shown. The color represents base-pairing probabilities.
